# Allosteric communication pathways routed by Ca^2+^/Mg^2+^ exchange in GCAP1 selectively switch target regulation modes

**DOI:** 10.1038/srep34277

**Published:** 2016-10-14

**Authors:** Valerio Marino, Daniele Dell’Orco

**Affiliations:** 1 Department of Neurosciences, Biomedicine and Movement Sciences, Section of Biological Chemistry, University of Verona, strada le Grazie 8, I-37134 Verona, Italy; 2 Centre for BioMedical Computing (CBMC), University of Verona, strada le Grazie 8, I-37134 Verona, Italy

## Abstract

GCAP1 is a neuronal calcium sensor protein that regulates the phototransduction cascade in vertebrates by switching between activator and inhibitor of the target guanylate cyclase (GC) in a Ca^2+^-dependent manner. We carried out exhaustive molecular dynamics simulations of GCAP1 and determined the intramolecular communication pathways involved in the specific GC activator/inhibitor switch. The switch was found to depend on the Mg^2+^/Ca^2+^ loading states of the three EF hands and on the way the information is transferred from each EF hand to specific residues at the GCAP1/GC interface. Post-translational myristoylation is fundamental to mediate long range allosteric interactions including the EF2-EF4 coupling and the communication between EF4 and the GC binding interface. Some hubs in the identified protein network are the target of retinal dystrophy mutations, suggesting that the lack of complete inhibition of GC observed in many cases is likely due to the perturbation of intra/intermolecular communication routes.

Conformational changes adopted by calcium-sensor proteins in response to Ca^2+^-binding allow them to selectively recognize and regulate their targets, thereby contributing to the control of several biological processes^1^. For some calcium sensors such as the ubiquitous calmodulin the conformational switch is substantial and associated to a high degree of structural plasticity, which allows the recognition and regulation of a variety of different targets^2^,^3^. Other Ca^2+^-sensors, however, are selectively expressed in specific cell types and regulate only a few, sometimes even unique biological targets. This is the case for the neuronal calcium sensors (NCS) family^4^,^5^.

Guanylate cyclase activating proteins (GCAPs) are NCS involved in the early steps of vertebrate vision, which shape the photoresponse of rods and cones under different light conditions, thus contributing to the second messenger-controlled recovery of the phototransduction machinery following light stimulation^6^. By binding up to three Ca^2+^ ions with high, submicromolar affinity and up to two Mg^2+^ ions with lower affinity in their three functional EF-hand motifs, GCAPs show a remarkable and rather unique regulatory capability. The same protein indeed acts as an inhibitor of the membrane bound guanylate cyclase (GC) at high (250–800 nM) intracellular [Ca^2+^] corresponding to dark-adapted conditions, while it switches to an activator of the enzyme at low (20–100 nM) [Ca^2+^], thereby stimulating the synthesis of the second messenger cGMP necessary to switch off the light-activated cascade^7^,^8^,^9^. Two to eight GCAP isoforms have been observed in species from fish to human and the role of different GCAPs in rods and cones is well described by a Ca^2+^-relay model, in which the control of photoreceptor physiological response depends on incremental changes in the cytoplasmic [Ca^2+^] sensed by GCAPs with slightly different affinity for Ca^2+^ 10,11,12.

GCAP1 is expressed both in rods and cones and has raised particular interest among other GCAPs as up to fifteen missense mutations in its codifying gene have been associated to retinal dystrophies^13^,^14^, a heterogeneous set of vision-threatening diseases often leading to complete blindness. Biochemical investigations revealed that the majority of GCAP1 molecules carrying the mutations exhibit a more or less severe disturbance in Ca^2+^-sensing properties, resulting in the constitutive activation of GC over the physiological range of [Ca^2+^], in which they normally switch between activating and inhibiting regulatory modes^15^,^16^,^17^,^18^,^19^,^20^. Some mutants have been shown to affect the binding of Ca^2+^ and Mg^2+^ to one of the three specific EF-hands in GCAP1^15^,^21^, however, in spite of the remarkable deregulation observed in GC activation, some other mutant showed no significant change in metal ion affinity compared to the wild type^18^.

Biochemical and biophysical investigations established that it is the selective binding of either Ca^2+^ or Mg^2+^ ions to specific EF-hands that triggers the switch of GCAP1 from its GC inhibitory (three Ca^2+^ ions bound to EF2, EF3 and EF4) to its GC activating form (one Mg^2+^ ion bound to EF2, and putatively to EF3)^7^,^8^,^9^. More recently, it was suggested that the covalent myristoylation at its N-terminal, which controls the affinity of the GCAP1-GC interaction^22^,^23^, is also involved in intramolecular communication with the opposite C-terminal domain by controlling the Ca^2+^ sensitivity of EF4^24^,^25^. A dynamic connection between the myristoyl moiety and EF4 was thus proposed, which implies a finely regulated allosteric communication between the N and C domains of GCAP1, however the mechanism by which Mg^2+^/ Ca^2+^ exchange determines the fate of GCAP1 as a Ca^2+^ sensor and GC-regulator remains largely unknown.

We present a thorough structural analysis of both myristoylated and nonmyristoylated GCAP1 based on exhaustive Molecular Dynamics (MD) simulations that unveiled intramolecular communication pathways involved in the specific switch between GC activator/inhibitor states. Our findings suggest that the binding of either Ca^2+^ or Mg^2+^ to specific EF-hands is crucial for routing the information transfer from the ion binding site to distal sites where the interaction with the target GC occurs. Some key residues appear to be especially important to structurally interconnect distal sites via robust allosteric pathways that are only partially overlapped in the activating and inhibitory states. Finally, we found that several residues that are target of retinal dystrophy-associated mutations constitute important intramolecular hubs for the communication routes in GCAP1 thus playing a particularly important role in maintaining its GC inhibitory state. Perturbation of specific intramolecular communication pathways likely results in a lack of complete inhibition of the target GC.

## Results

### Exhaustive and convergent conformational sampling of GC-inhibiting and activating states of GCAP1 by Molecular Dynamics simulations

Myristoylated and nonmyristoylated GCAP1 (mGCAP1 and nmGCAP1, respectively) were simulated in their GC-inhibiting and activating states. Several independent studies (see Ref. 10 for a review) confirmed that the maximal GC inhibition by GCAP1 is achieved when each canonical EF hand is occupied by a Ca^2+^ ion. We therefore indicate the GC-inhibiting state as EF2^Ca^EF3^Ca^EF4^Ca^ throughout this paper. The GC-activating state is instead associated to GCAP1 with a Mg^2+^ ion bound in EF2, and possibly in EF3 too^7^,^8^,^9^,^26^. We therefore indicate these activating states as EF2^Mg^ and EF2^Mg^EF3^Mg^, respectively.

**Figure 1:**
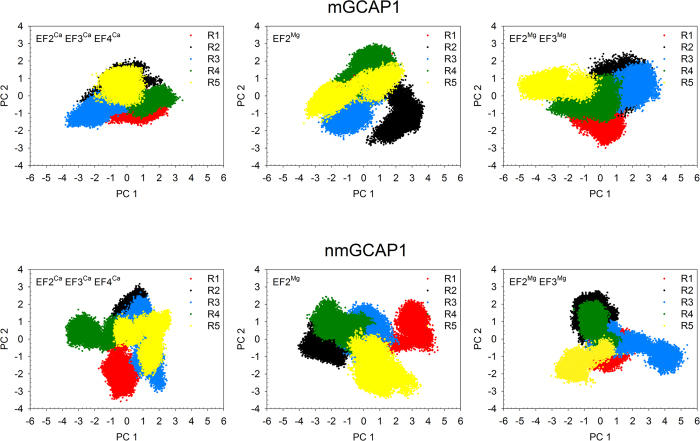
Projections of the frames extracted from each of the five 200 ns MD simulation replicas (R1 to R5) along the first two Principal Components (PC1 and PC2) calculated on the concatenated 1 μs trajectories of mGCAP1 (top panels) and nmGCAP1 (bottom panels) in their EF2^Ca^EF3^Ca^EF4^Ca^ (left), EF2^Mg^ (center) and EF2^Mg^EF3^Mg^ (right) forms. R1 frames are represented in red, R2 frames are black, R3 frames are blue, R4 frames are green, R5 frames are yellow.

Five replicas of 200 ns MD simulations were run for each GCAP1 state and results were considered both individually and by concatenating the trajectories to form a comprehensive 1 μs trajectory. Root-mean square deviation plots calculated on Cα-atoms (RMSD) with respect to the equilibrated structures for the five 200 ns replicas of each simulated state are reported in Supplementary Figure S1. RMSD average values ranged between 2.18 ± 0.29 Å and 2.95 ± 0.67 Å, indicative of good reproducibility across each replica. However, it is well established that independent MD trajectories of the same system at the same temperature may sample slightly different portions of the conformational landscape, therefore RMSD *per se* is not an accurate index for sampling convergence^27^. We therefore performed Principal Component Analysis (PCA) of Cα fluctuations on each individual trajectory in order to define a low-dimensional subspace, in which essential protein dynamics can be better described. Projections of the MD trajectory from each replica along the first two principal components (PC1 and PC2) are shown in Fig. 1. The substantial overlapping of the scatter plots in the PC space sampled is consistent with a homogeneous sampling for each replica.

**Figure 2:**
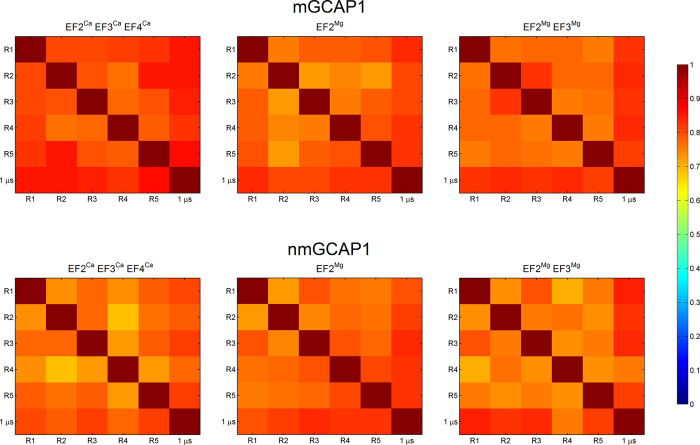
Root-Mean Square Inner Product (RMSIP) of the first 20 Principal Components representing the low frequency motions of each of the five 200 ns MD simulation replicas (R1 to R5) of mGCAP1 (top panels) and nmGCAP1 (bottom panels) in their EF2^Ca^EF3^Ca^EF4^Ca^ (left), EF2^Mg^ (center) and EF2^Mg^EF3^Mg^ (right) forms. The RMSIP is calculated for each replica against each other and against the concatenated trajectory (1 μs) and is represented in a color scale from blue (0) to dark red (1).

The similarity and therefore consistency between the conformational subspaces sampled by each replica and, comprehensively, by the concatenated trajectory was probed by computing the Root-Mean-Square Inner Product (RMSIP) of the first 20 PC, representing eigenvectors of the covariance matrix of each replica or of the whole concatenated trajectory (Fig. 2). Satisfactory values were obtained in all cases as proven by RMSIP values approaching unity both when comparing each replica with one another and when the comparison was extended to the concatenated comprehensive 1 μs trajectory. RMSIP values for nonmyristoylated GCAP1 were on average smaller compared to the myristoylated case, however they did not decrease below 0.65 (Fig. 2).

A further proof of conformational sampling convergence was provided by the computation of the cosine content *c*_*1*_ of the first PC (PC1), which represents a necessary condition for the sampling convergence. It was demonstrated that insufficient sampling could lead to high (close to unity) *c*_*i*_ values, where *i* refers to any PC_*i*_^27^,^28^. The *c*_*1*_ of the six concatenated trajectories of GCAP1 states ranged between 0.0008 and 0.4246, while the average *c*_*1*_ of the five 200 ns replicas for each simulated GCAP1 state ranged between 0.2490 and 0.6873. Since *c*_*1*_ relative to the 1 μs concatenated trajectories were found to be lower than the average *c*_*1*_*’s* of each replica, the conformational sampling achieved when the concatenated trajectory was considered was generally significant and satisfactory.

Overall, these results confirm that each 200 ns MD replica represents an independent and conformationally consistent dynamic description of GCAP1 in all the tested states, and that the concatenated 1 μs trajectories constitute a suitable framework where to exhaustively investigate essential dynamic properties.

### Persistent noncovalent interactions along MD trajectories of GCAP1 define a network with specific hubs and intramolecular communication routes

Hydrophobic, electrostatic and H-bond interactions between residues within the protein milieu may be highly transient or significantly persist in time, when they are aimed at maintaining a noncovalent contact necessary to transfer the information intramolecularly. Monitoring the time course of noncovalent interactions along the concatenated 1 μs MD trajectory of GCAP1 hence allowed us to eventually determine whether and how long would contacts persist between groups of interactions, in which amino acids are nodes and interactions are edges or arches in a graph representing the Protein Structure Network (PSN)^29^,^30^,^31^.

**Figure 3:**
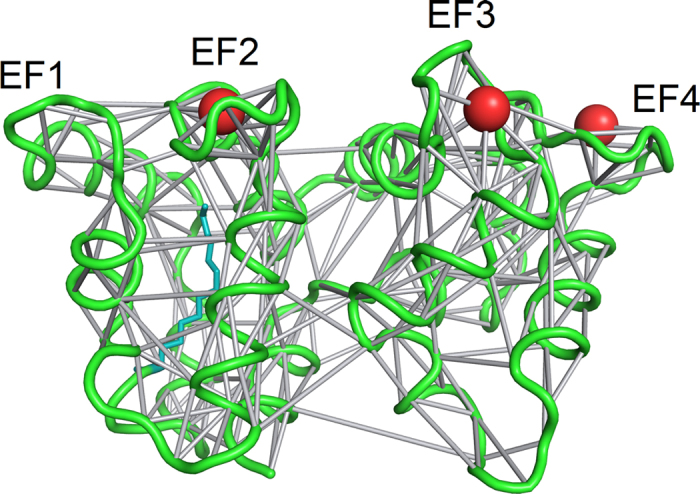
Example of Protein Structure Network calculated with PyInteraph for 1 μs MD simulation of EF2^Ca^EF3^Ca^EF4^Ca^ mGCAP1. Secondary structure is represented in green cartoons, Ca^2+^ ions are shown as red spheres, the myristoyl group is represented by teal sticks, edges representing persistent interactions between residues (nodes) are shown as grey sticks connecting Cα-atoms or ions.

**Figure 4:**
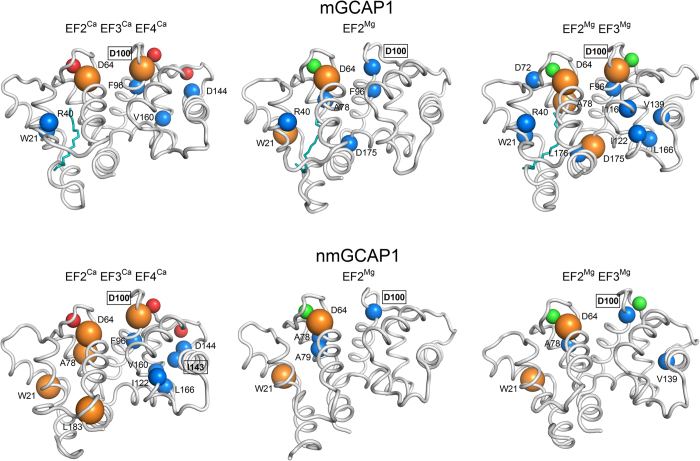
Highest degree hubs identified by PyInteraph in the PSN of mGCAP1 (top panels) and nmGCAP1 (bottom panels) in their EF2^Ca^EF3^Ca^EF4^Ca^ (left), EF2^Mg^ (center) and EF2^Mg^EF3^Mg^ (right) forms. Secondary structure is represented in grey cartoons, Ca^2+^ ions are shown as red spheres, Mg^2+^ ions are shown as green spheres, the myristoyl group is represented as teal sticks, Cα of degree 7 hub residues are represented in blue spheres, Cα of degree 8 hub residues are represented in orange spheres with increased radius. Residues whose mutation is associated with cone, cone-rod or macular dystrophies are labelled in bold and framed.

The dynamic PSN calculated with the software PyInteraph (see Methods) over 1 μs MD simulations of EF2^Ca^EF3^Ca^EF4^Ca^ mGCAP1 is shown in Fig. 3 as an example. The analysis highlighted the presence of some amino acids and ions forming persistent contacts with many neighboring residues, thus constituting hubs for the network. Persistence thresholds (p_T_) for each simulated GCAP1 state were estimated (see Methods) in order to filter out transient interactions between residues, and ranged between 19.5% and 28.2%. Persistent interactions represented by edges in the PSN graph connect nodes located in even distal positions within GCAP1 tertiary structure. A thorough analysis of the degree of each hub, representing the number of neighboring residues per hub, as well as its position within the GCAP1 fold revealed important features for mGCAP1 and nmGCAP1 specific for each GC regulatory state. The highest degree hubs (degree = 7 or 8) in EF2^Ca^EF3^Ca^EF4^Ca^ mGCAP1 are located in both the N- and C-terminal domains (Fig. 4, top), while they mostly concentrate in the C-domain in the EF2^Mg^ mGCAP1 state. The EF2^Mg^EF3^Mg^ putative GC activating state of mGCAP1 showed a broader distribution, with 13 high-degree hubs compared to the 7 ones in the EF2^Ca^EF3^Ca^EF4^Ca^ and EF2^Mg^ mGCAP1 states (Supplementary Table T1 and Fig. 4, top). Interestingly, D64 and D100, respectively the first Ca^2+^-coordinator residues of EF2 and EF3 were found among the highest-degree hubs in all mGCAP1 states, while other high-degree hubs varied depending on the GC regulatory state (Fig. 4, Supplementary Table T1). While the same two residues constituted high-degree hubs also for nmGCAP1 in all the three tested GC regulatory states (Supplementary Table T2 and Fig. 4, bottom), the GCAP1 variant lacking the myristoyl modification showed many more high-degree hubs (11 vs. 7) in the EF2^Ca^EF3^Ca^EF4^Ca^ state, and less in the EF2^Mg^ and EF2^Mg^EF3^Mg^ states (5 hubs for nmGCAP1 versus 7 and 13 for mGCAP1, respectively; Supplementary Tables T1 and T2 and Fig. 4).

Interestingly, the analysis of PSN hubs with a degree down to 4 highlighted for both mGCAP1 and nmGCAP1 the presence of several residues target of retinal dystrophies, especially in the EF2^Ca^EF3^Ca^EF4^Ca^ GC-inhibitory state (Supplementary Tables T1 and T2). On the contrary, the myristoyl moiety was never found among the high-degree hubs (Supplementary Table T1), indicating the presence of many hydrophobic, yet non-persistent interactions along the 1 μs simulations.

An alternative PSN analysis performed using the elastic network analysis implemented in the WebPSN software (see Methods) overall suggested a similar role for the most important hubs already found by the dynamic analysis (Supplementary Table T3), although the consistency was in general not high.

### Intermolecular communication between GCAP1 and its target: information transfer from each EF hand to the putative GC/GCAP1 binding interface

The conformational switch of GCAP1 between GC activating/inhibiting states following the specific exchange of metal ions in its EF-hands must be communicated to the target via a specific protein-protein interface. Several groups used different techniques to determine the putative GCAP1/GC interface^32^,^33^,^34^ and consistently identified specific regions in EF1 and in the exiting helix of EF2 as the main constituents of the binding interface^32^,^33^,^34^. In addition, other residues located in the entering helix of EF3 have been shown to be important for GC binding and activation^32^,^34^.

**Figure 5:**
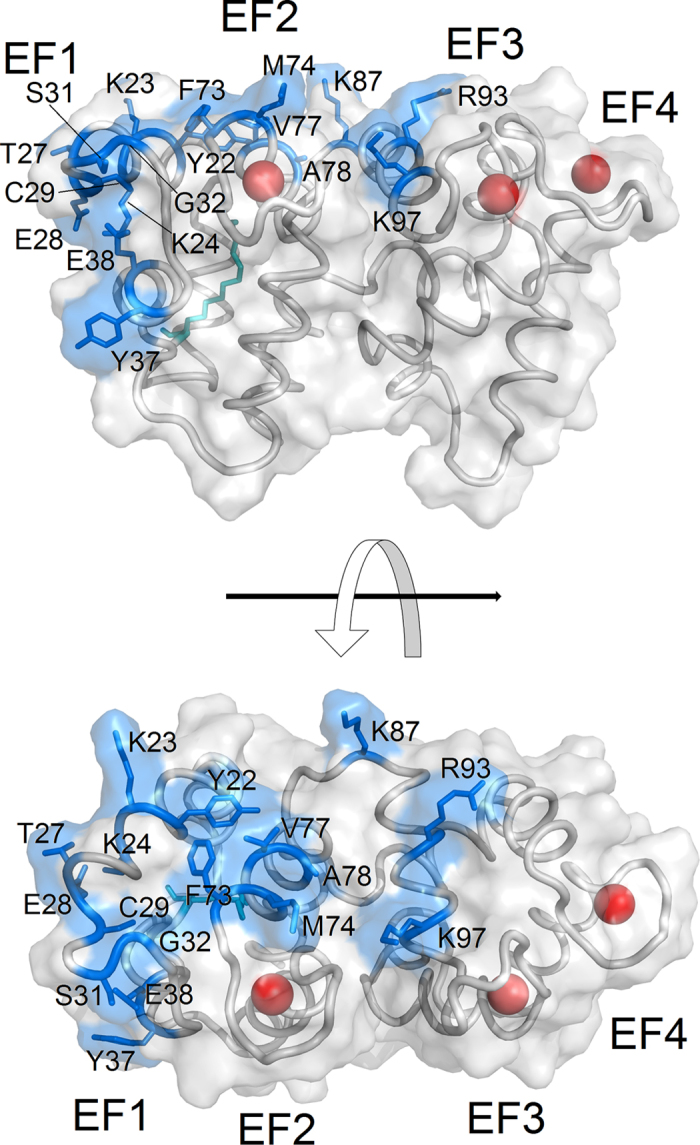
Residues of mGCAP1 involved in the putative interface with the target Guanylate Cyclase. The EF2^Ca^EF3^Ca^EF4^Ca^ form is represented in grey cartoons together with its molecular surface, Ca^2+^ ions are shown as red spheres, the myristoyl group is represented in teal sticks, residues belonging to the putative GC interface are represented as blue sticks together with their molecular surface. Residues forming the putative GCAP1/GC interface have been identified in former studies (see text and refs 32,33,34).

We considered all the residues suggested to form the putative GCAP1/GC binding interface, which is represented in Fig. 5. Overall, the identified interface shows somewhat disconnected areas, which seems *prima facie* incompatible with a unique GCAP1/GC binding mode. However, it might be possible that the interface follows the conformational switch of GCAP1 between its GC inhibiting/activating states, thereby plastically remodeling itself to cover specific regions in the different regulatory states. We therefore explicitly considered all plausible binding sites between GCAP1 and GC and investigated how the information may route into specific pathways interconnecting each EF-hand to each putative GC binding site.

**Figure 6:**
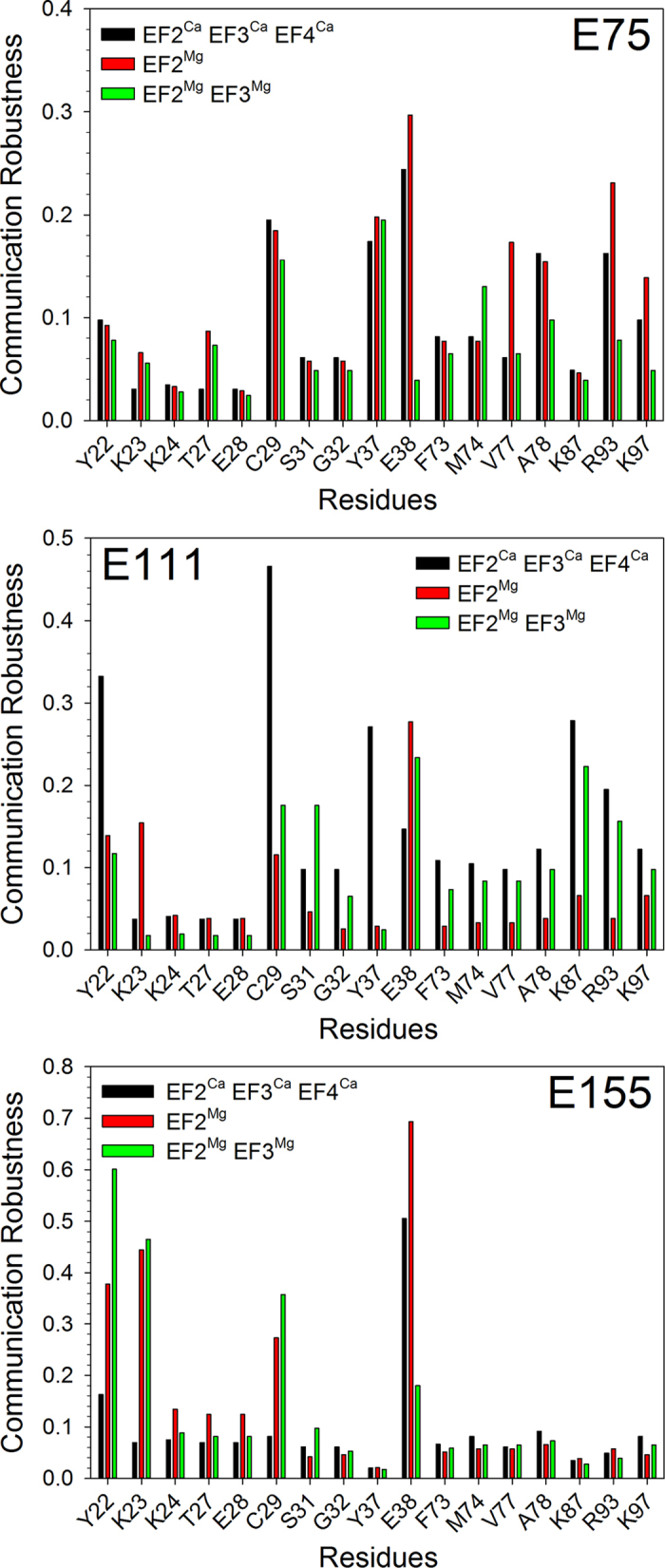
Communication Robustness between bidentate Glu residues of EF2 (top), EF3 (middle), EF4 (bottom) and residues belonging to the GC binding interface of mGCAP1 in its EF2^Ca^EF3^Ca^EF4^Ca^ (black), EF2^Mg^ (red) and EF2^Mg^EF3^Mg^ (green) forms.

In order for a communication pathway connecting two nodes A and B to be robust, some requirements should be fulfilled. According to the parsimony principle, the higher the number of shortest paths connecting A and B and the more persistent the noncovalent interactions defining the path itself, the more robust the communication pathway. We translated these requirements into a simple definition of the communication robustness (CR) index defined in Equation 1:


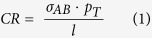


where *σ*_*AB*_ indicates the number of paths with the shortest length connecting A and B, *p*_*T*_ is the significance threshold for the interaction persistence (see Methods) and *l* is the length of the shortest paths. The CR index defined above allowed us to systematically investigate the robustness of all identified pathways interconnecting each EF hand, where the Ca^2+^/Mg^2+^ binding occurs, with each putative GC binding site, in all the GC regulatory states of GCAP1. As a residue representative for the corresponding EF hand we chose to consider the glutamate providing the bidentate oxygen ligands for Ca^2+^, namely E75 for EF2, E111 for EF3 and E155 for EF4. Results of the analyses for mGCAP1 are reported in Fig. 6.

As a general trend, no particularly high-robustness pathway for GC regulation was found starting from EF2 (E75) considering the relatively short distance between the Ca^2+^/Mg^2+^ binding region and most of the GC putative binding residues (Fig. 5). The computed CR index indeed never exceeded 0.3, the value observed for E38 in the EF2^Mg^ state, and no major difference was observed for the tested mGCAP1 regulatory states (Fig. 6). In contrast, the highest affinity binding site for Ca^2+^ E111 (EF3) resulted to be more robustly connected to GC interfacial residues, especially in the EF2^Ca^EF3^Ca^EF4^Ca^-GC inhibiting state, mostly via the Y22, C29, Y37 and K87 residues (Fig. 6). Switching to GC activating states, either EF2^Mg^ or EF2^Mg^EF3^Mg^ significantly decreased the communication robustness, except for E38, which showed an opposite trend, where CR increased from 0.14 to 0.24–0.26. E38 thus seems to be an essential residue to mediate target inhibition by ensuring the communication of the Ca^2+^-loaded state of EF3. Interestingly, the relatively high distance between EF4 (E155) and all putative GC binding sites did not prevent the emergence of some particularly robust communication pathways (Fig. 6). While generally E155 showed a low CR index (≤0.1) for many GC interfacial residues, four of them showed remarkably high robustness, namely E38 in its EF2^Ca^EF3^Ca^EF4^Ca^ (CR = 0.5) and EF2^Mg^ (CR = 0.7) states, Y22, K23 and C29 in the GC activating states (EF2^Mg^ or EF2^Mg^EF3^Mg^; CR values between 0.3 and 0.6). The particularly high CR values in the Mg^2+^-bound states for Y22 and K23 suggest that these two residues are important for communicating to the GC the unloaded status of EF4. In general, the metal-bound state of the distal EF4 motif in mGCAP1 has to be therefore precisely and robustly communicated to specific GC binding residues to ensure the target switch between its operational states.

Interestingly, only a few residues forming the GC binding interface show robust intramolecular communication with all three EF hands in each GC regulatory state. E38, located at the exiting helix in the EF1 motif (Fig. 5) seems to be well connected to both EF2 (CR between 0.25 and 0.30 with E75 in the EF2^Ca^EF3^Ca^EF4^Ca^ and EF2^Mg^ states, respectively) and EF3 (CR 0.16–0.28 with E111), and as noticed extremely well connected to EF4, giving the highest CR index in this case (CR 0.5–0.7 with E155) (see Fig. 6). Therefore, E38 seems to be an essential residue to communicate to the target GC about the loading status of the distal EF-hand 4. Particularly apparent was also the robustness of the communication pathways found between EF3 and C29 (Fig. 6), located in the entering helix in EF1 not far from E38, however robustness was limited to the EF2^Ca^EF3^Ca^EF4^Ca^ state (CR = 0.48 vs. 0.12 in the EF2^Mg^ state). This could imply that the contact between GC and C29 is necessary to keep the target in its inhibiting state. Other specific patterns of less robust communication could be observed between each EF hand and GC interfacial site (Fig. 6).

**Figure 7:**
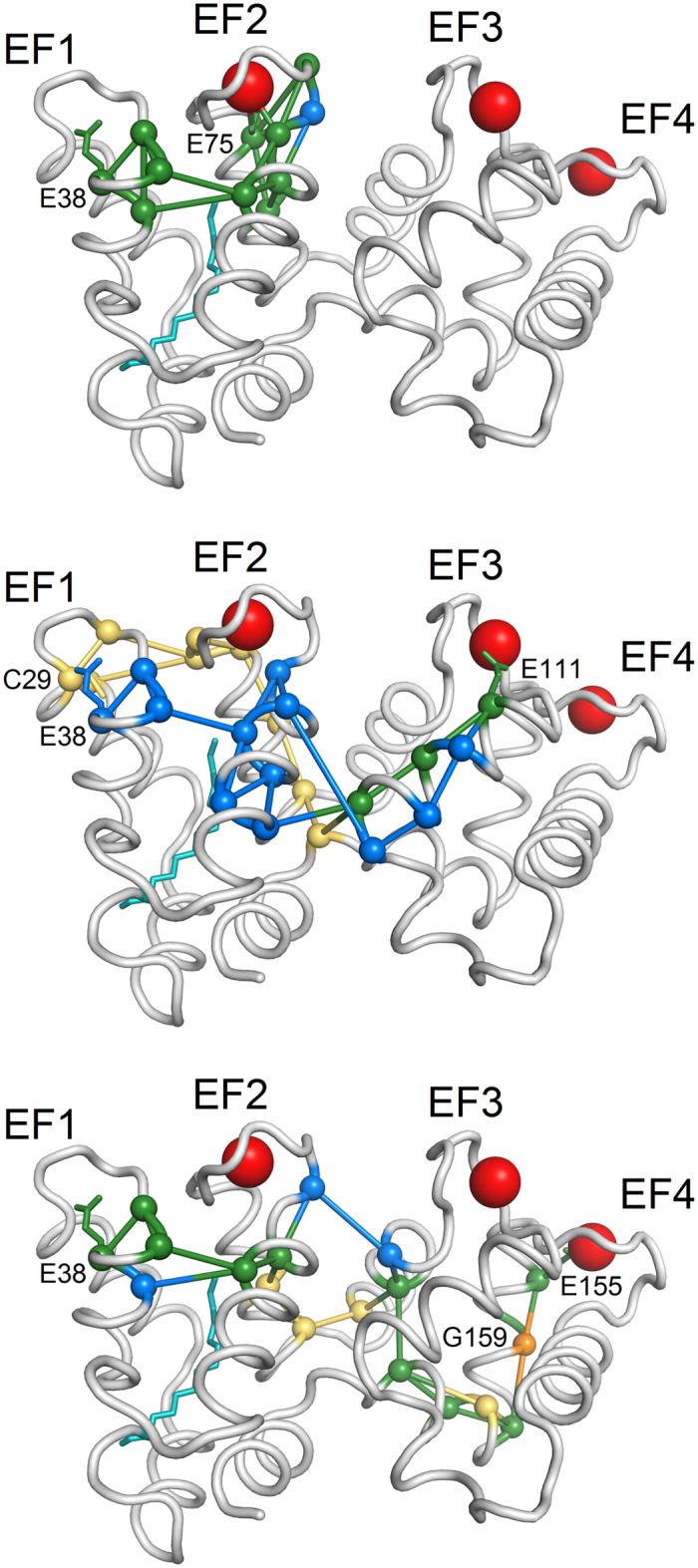
Paths constituted by nodes with the highest selective betweenness connecting bidentate Glu residues of EF2 (top), EF3 (middle) and EF4 (bottom) and the residues belonging to the GC binding interface with the highest CR. Secondary structure is represented in grey cartoons, Ca^2+^ ions are shown as red spheres, the myristoyl group is shown as teal sticks, Cα of the residues belonging to the paths are shown as spheres, edges are shown as sticks, the sidechains of the extremes of the paths are represented as sticks. Nodes and edges specific of the EF2^Ca^EF3^Ca^EF4^Ca^ form are represented in yellow, those specific of the EF2^Mg^ form are blue, those in common are green. Residue G159, whose mutation in V is associated with retinal dystrophies, is represented in orange.

The dynamic PSN analysis further allowed us to determine for each amino acid (or node) the selective betweenness centrality (SB, see Methods), indicative of the number of shortest paths between specific vertices that pass through that node. Figure 7 and the Supplementary Videos V1, V2 and V3 show the pathways identified by nodes with the highest SB connecting each EF hand bidentate ligand with the residue in the putative GC binding interface, for which the CR was maximal. As seen above (Fig. 6), EF2 (E75 residue) was mostly connected to E38 (Fig. 7, top) in both GC activating (blue path) and inhibiting (yellow path) states, while EF3 (E111 residue) showed higher robustness in communication with C29 (Fig. 7, middle) in the GC inhibiting state (yellow path) and with E38 in the GC activating state (blue path). Finally, EF4 (E155 residue) was robustly connected to E38 (Fig. 7, bottom) in both the GC activating (blue path) and inhibiting (yellow path) states. It is worth noting that the individuated communication pathways can be partly overlapped (green paths), meaning that some residues are important both for transferring the information to the proper GC binding site in the GC activating and inhibiting states of GCAP1 (Fig. 7).

### Intramolecular communication between EF hands in mGCAP1

**Figure 8:**
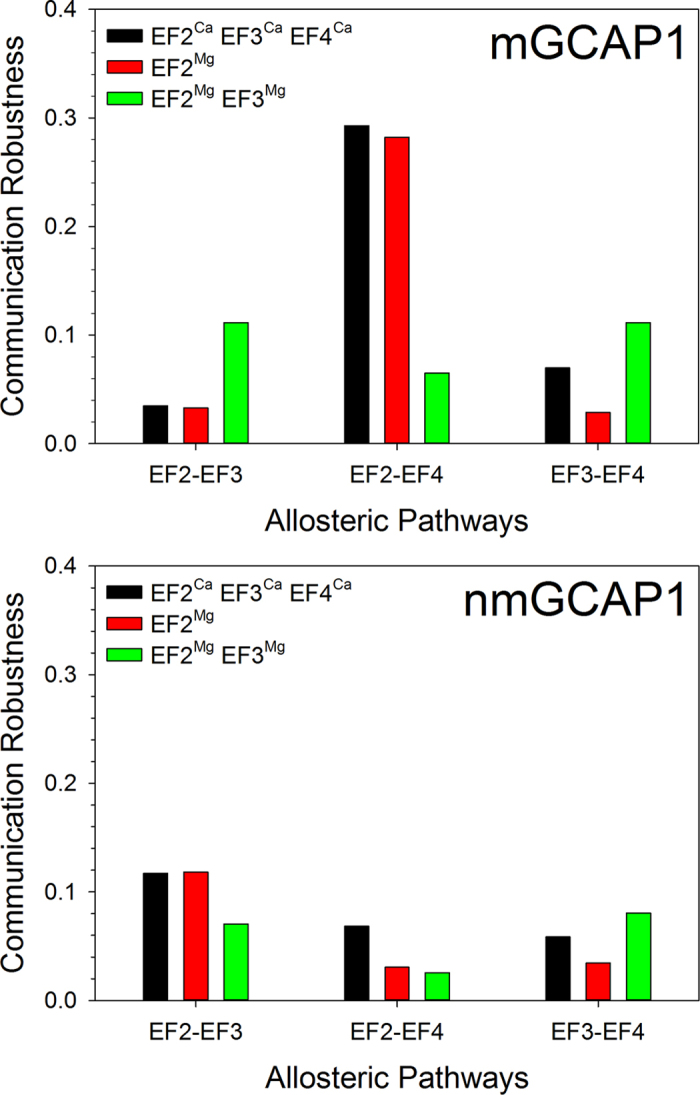
Communication Robustness among bidentate Glu residues of EF2, EF3 and EF4 of mGCAP1 (top) and nmGCAP1 (bottom) in their EF2^Ca^EF3^Ca^EF4^Ca^ (black), EF2^Mg^ (red) and EF2^Mg^EF3^Mg^ (green) forms.

The analysis of intramolecular communication in GCAP1 in its various GC regulatory states was performed also to unveil potential allosteric mechanisms involving the metal ion-binding sites. We therefore calculated the CR index along the 1 μs MD trajectory between each bidentate ion ligand in EF2, EF3 and EF4. Results for mGCAP1 are shown in Fig. 8 (top). No significantly robust communication pathway could be identified that connects EF2 with the contiguous EF3 or EF3 with EF4. Surprisingly, a significantly high CR (approximately 0.3) was detected for the connection between EF2 and EF4, both in the EF2^Ca^EF3^Ca^EF4^Ca^-GC inhibiting and EF2^Mg^-GC activating states. The connection lost robustness in the EF2^Mg^EF3^Mg^ state (CR ≈ 0.06; Fig. 8, top).

### Role of myristoyl in mediating allosteric communications and GC interactions

The GCAP1 form lacking the myristoyl moiety showed a somewhat different pattern of intramolecular communication robustness concerning the connections between EF hands and putative GC binding sites (Supplementary Figure S4). While a general trend of decreased robustness could be observed in all the tested GCAP1 states (compare Fig. 6 and Supplementary Figure S4), the most apparent differences are the following: a) an almost complete loss of robust communication pathways between EF4 (E155) and the putative GC-binding sites was found for nmGCAP1 (Supplementary Figure S4, bottom), the only exceptions being the mildly robust communication with E38 and G32 in the EF2^Mg^EF3^Mg^ state; b) the highly robust communication pathways identified between EF3 (E111) and some GC-interface residues in the GC-inhibiting state were replaced by some lower robustness communication pathways with G32, Y37 and F73 (Supplementary Figure S4, middle) in the EF2^Mg^, GC-activating state; c) the interaction between E38 and EF2 (E75) remained highly robust for nmGCAP1 (CR = 0.32) only in the EF2^Ca^EF3^Ca^EF4^Ca^-GC inhibiting state, at odds with the myristoylated form, for which both GC regulatory states had robust connections (Supplementary Figure S4, top, vs Fig. 6, top).

Significant differences could be observed for nmGCAP1 compared to the myristoylated form also regarding the inter EF-hand communication (Fig. 8, bottom). Interestingly, no significantly robust communication was observed between either couple of EF-hands in each tested GC regulatory state for nmGCAP1, in particular the distal robust communication between EF2 and EF4 observed for mGCAP1 in both GC activating and inhibitory states was completely lost (Fig. 8, bottom).

We also checked whether robust communication pathways could be identified between the myristoyl moiety and the putative GC binding site. Results reported in Supplementary Figure S2 (top) showed that this is not generally the case. Indeed, except for M74 in the EF2^Ca^EF3^Ca^EF4^Ca^ and EF2^Mg^EF3^Mg^ states (CR ≈0.18), R93 in both Mg^2+^-bound states (CR≈0.14) and Y37 in the EF2^Ca^EF3^Ca^EF4^Ca^ state (CR ≈0.12) all other residues were not connected to the myristoyl by robust pathways. Mildly robust communication pathways were detected between the myristoyl and EF2 in the EF2^Ca^EF3^Ca^EF4^Ca^ state (CR ≈0.25), however CR was halved in both Mg^2+^-bound states (Supplementary Figure S2, bottom). Except for the GC-inhibiting state, for which a significant CR was detected for the communication with EF3 (E111) no other state showed significant values for the communication robustness, in particular no robust path could be identified that connects the myristoyl moiety with EF4 (Supplementary Figure S2, bottom).

## Discussion

GCAPs share their general three-dimensional fold with the other members of the NCS family. However, they show the rather unique feature of being at the same time activators and inhibitors of their biological GC target depending on the level of intracellular calcium. Understanding the mechanism by which relatively small protein conformational changes result in such diverse biochemical properties is not trivial, and yet it would be crucial to shed light on the molecular basis of some inherited retinal diseases caused by the deregulation of second messenger homeostasis in photoreceptors. This is especially true for GCAP1, for which up to 15 point mutations have been found to date that cause cone, cone/rod and/or macular dystrophies^13^,^14^,^15^,^16^,^17^,^18^,^19^,^20^.

We performed exhaustive MD simulations of both myristoylated and nonmyristoylated GCAP1 in each GC regulatory state. A comprehensive and consistent sampling of the conformational space has been achieved by the analysis of protein essential dynamics (Figs 1 and 2), which focuses on collective motions to unveil protein conformational transitions^35^. Previous MD studies by us^8^,^36^ and others^37^ as well as dedicated biophysical experiments^38^,^39^ showed that the Ca^2+^ binding induces relatively small conformational changes in GCAP1, and that its replacement with Mg^2+^ in specific binding sites may significantly perturb protein compactness and flexibility^8^. In the present study we investigated the details of GCAP1 structural dynamics by carrying out a set of more extensive MD simulations. We concatenated the trajectories from five 200 ns replicas of GCAP1, thus ending up in a consistent 1 μs trajectory where we could perform network-level analyses and highlight long-range communication pathways. The PSN paradigm applied to sufficiently long and convergent MD trajectories has a great potential to find out connections between conformational dynamics and allostery mechanisms, as it has been shown recently in other protein systems^30^,^40^.

An important finding in our study is that the Mg^2+^/Ca^2+^ exchange in specific EF hands creates selective intramolecular pathways connecting directly the metal ion binding sites with the GC binding sites. According to the PSN paradigm, in which amino acids are nodes and the interaction among them edges, some residues acquire the role of hubs for they mediate many persistent interactions (Figs 3 and 4). The number and the distribution of high degree hubs was shown to depend on the specific GC regulating state and the presence or absence of the myristoyl modification (Fig. 4 and Supplementary Tables T1-T3). The dynamic analysis performed with PyInteraph highlighted the constant presence of D64 and D100 among the highest-degree hubs, a particularly important result since D100 is the target of the cone-dystrophy related D100E/G mutations^17^,^41^. Other residues target of retinal dystrophy-associated point mutations were found among high-degree hubs both in the GC activating and inhibiting states of mGCAP1 and, to a lower extent, of nmGCAP1 (Supplementary Tables T1 and T2). Partly consistent results were found when the elastic network/PSN analysis was carried out with the WebPSN tool (Supplementary Table T3). Some level of discrepancy with the results obtained by PyInteraph is indeed not surprising, as WebPSN is based on a simple armonic potential applied to a static starting structure, therefore the approach keeps a memory of such initial conditions. Nonetheless, the method suggested a similar role for the most important hubs (Supplementary Table T3).

The communication pathways in GCAP1 are determined by the persistent contacts between contiguous amino acids, which define specific routes extending within the protein structure. These selective routes stabilize specific interaction patterns that are necessary for achieving GC inhibition or activation. This concept may help solving the puzzling evidence of disconnected GC/GCAP1 interfacial areas observed in independent studies^32^,^33^,^34^. According to the Mg^2+^/Ca^2+^ loading status of each EF-hand, specific communication routes are created within GCAP1 structure that end up in appropriate residues to communicate and trigger GC activation or inhibition. As a consequence, the GCAP1/GC interface might therefore “plastically” adapt to the specific GCAP1 status in order to optimize the protein-protein contacts for proper information transfer. The close proximity of such residues (Fig. 5) would allow the interface optimization to occur without major conformational changes.

Our network-level analysis permitted the identification of specific long range communication pathways connecting distal GCAP1 regions belonging to the N- and C-terminal domains, and to assess the role of myristoylation in mediating such interactions. In spite of the relatively long distance from the putative GC binding sites, EF4 has been shown to robustly communicate with some residues at the GC interfacial region, including the spatially close E38 and C29 (Fig. 5), which are also the ending points of very robust intramolecular routes originated in EF3 and, to a lower extent, EF2 (Figs 6 and 7). Pathways made up of nodes with the highest SB route the information transfer to close or identical interfacial residues of mGCAP1 in its different GC regulatory states, however they are only partly overlapped (Fig. 7). The pattern of intramolecular communication pathways was shown to be considerably different for nmGCAP1 (Supplementary Figure S4). In general, no significantly robust communication was observed in this case between EF4 and the GC putative binding sites (Supplementary Figure S4, bottom). The myristoyl group was never found among the highest-degree hubs (Supplementary Tables T1-T3), and its communication robustness with the GC putative binding sites was modest in each simulated mGCAP1 state (Supplementary Figure S2, top). The fact that the communication robustness with GCAP1 EF hands was found to be significant only for the nearby EF2 in its GC-inhibiting state (Supplementary Figure S2, bottom) suggests that the myristoyl is a transient connector of important intramolecular interactions rather that a central hub. Its presence is however essential to guarantee the correct routing of the information transfer about the loading state from EF4 to the GC binding site. This finding is substantially in line with the “tug” mechanism proposed by Peshenko *et al*.^25^, in which a dynamic connection between the myristoyl and EF4 was hypothesized to attenuate the efficiency of GC activation in exchange for optimal Ca^2+^ sensitivity. The importance of Ca^2+^ binding to EF4 in propagating the conformational information to the GC binding interface, thus leading to the inhibiting/activating switch was recently highlighted by Lim *et al*. in an NMR study, in which the largest Ca^2+^-dependent structural differences were observed in the EF4 region and in the adjacent so called “switch helix” composed by the 164–174 stretch^24^. Our results are fully consistent with this observation, as all the pathways constituted by nodes with the highest SB connecting EF4 and GC putative binding interface residues include residues belonging to the switch helix (Fig. 7). Our study thus confirms the role of this helical stretch, which may act as a conduit relaying Ca^2+^-induced structural changes in EF4 to the GC binding site^24^.

Another important result from the dynamic analysis performed here is that a robust long range communication between EF2 and EF4, which are distal metal ion binding motifs belonging to the N- and C-terminal domains respectively, was clearly detected both for the GC activating and inhibiting state of mGCAP1 (Fig. 8). This interdomain allosteric interaction takes place only in the presence of the N-terminal myristoylation (Fig. 8), and is likely crucial for GCAP1 switch function as it connects the two metal ion binding sites with intermediate (EF4) and lowest (EF2) affinity for Ca^2+^, which have respectively negligible and highest affinity for Mg^2+^ 7,9. It has been long established that the Ca^2+^-mediated activation of GC by GCAPs is highly cooperative^42^ although biophysical evidence strongly suggests that Ca^2+^ binding to GCAP1 occurs without any apparent cooperativity^15^,^43^, except for one case (L84F mGCAP1) in which cooperativity was observed^18^. Our data show that an allosteric mechanism may connect two metal ion binding sites without observed cooperative effects on the binding affinity. The presence or the absence of a specific ion bound to an EF hand can then be communicated to a distal EF hand without affecting the metal ion affinity of this latter.

In conclusion, our analysis suggests that GCAP1 capability of switching between different GC regulatory states depends on the Mg^2+^/Ca^2+^ binding states and the way the information is transferred to specific residues at the interface with the target. Post translational myristoylation is fundamental to mediate long range allosteric interactions although the fatty acid *per se* is not a hub for intramolecular interactions. Some amino acids with high selective betweenness centrality in the PSN may largely perturb the intramolecular communication pathways necessary for switching between GCAP1 regulatory modes, and the fact that some hubs are target of retinal dystrophy mutants suggests that the lack of complete inhibition of the target GC observed in many cases is likely due to the perturbation of these intermolecular routes. More in general, our computational approach can be applied to other NCS proteins and extended to investigate intermolecular as well as intramolecular communication, thus being of great potential for unveiling poorly understood molecular mechanisms under physiological and altered conditions.

## Methods

### Molecular Dynamics simulations of GCAP1 in its GC activating/inhibiting states

The homology model of human mGCAP1 in its EF2^Ca^EF3^Ca^EF4^Ca^ state was built using the structure of chicken mGCAP1^44^ as template using a previously elucidated protocol^15^. Models of nmGCAP1 were built by removing the myristoyl group, EF2^Mg^ and EF2^Mg^EF3^Mg^ m/nmGCAP1 states were modeled either by removing Ca^2+^ ions from the respective EF-hand or by replacing them with Mg^2+^ ions as reported in ref. 8.

MD simulations of all GCAP1 states were performed using GROMACS 5.0.4 simulation package^45^ with the CHARMM27 all-atom force field^46^. All settings for MD simulation and CHARMM27 parameters for the myristoyl group were the same as in ref. 8, in which details are provided. All structures were subjected to energy minimization, first with the steepest descent (F_max_ = 1000 kJ mol^−1^ nm^−1^), then with the conjugate gradients (F_max_ = 500 kJ mol^−1^ nm^−1^) algorithm, by keeping the position of backbone atoms and ions fixed in both cases. The systems were equilibrated at 310 K for 2 ns of backbone position-restrained MD simulations and then at 310 K for 2 ns of unrestricted MD simulations. After equilibration, all systems underwent 200 ns unrestrained isothermal-isobaric (NPT ensemble, T = 310 K, P = 1 atm) MD simulation. To achieve an exhaustive sampling of the conformational space, five independent replicas of each system were generated by changing the random seed for the generation of initial velocities of the two equilibration and the production phases.

In order to assess the stability of the simulations, the RMSD for the five 200 ns replicas of each state was calculated on Cα atoms with respect to the relative structure after 4 ns equilibration. The average RMSD values ranged between 2.18 and 2.95 Å in the tested GCAP1 states.

### Principal Component Analysis

PCA is a widely-known tool to filter high-frequency motions in MD trajectories, taking advantage of the eigenvectors and eigenvalues of the mass-weighted covariance matrix (C) of the atomic positional fluctuations^47^. The C matrix was calculated for each state on protein alpha-carbons (Cα) of each replica and of the concatenated trajectories after superimposition to a reference structure, which was chosen as the conformation observed after the 4 ns equilibration phase in one of the five different replicas.

The PCA technique is based on the diagonalization of matrix C allowing for the identification of a set of eigenvectors and eigenvalues describing, respectively, direction and amplitude of the collective atomic motions or principal components (PC). Eigenvalues are ranked in decreasing order, therefore PC1 is the direction which describes the largest collective motion of the system, PC2 accounts for the second largest motion and so on. In order to analyze the consistency of the conformational space sampled by each replica, we projected the simulated trajectories on the first two PCs for the comparison of the main collective motions (Fig. 1).

It is possible to define the Essential Subspace (ES) as a subset of PCs which accounts for a percentage of total motion, in order to remove the noise represented by high frequency motions. Such percentage can be computed as the ratio between cumulative eigenvalues relative to the eigenvectors belonging to the ES and the sum of all eigenvalues of the system. The ES was defined by the first 20 PC of each replica and concatenated trajectories, describing at least 80% of the variance.

The sampling convergence of each individual MD replica as well as the concatenated trajectories was assessed by computing both the cosine content and the RMSIP indexes for each GCAP1 simulated state. The cosine content (*c*_*1*_)^48^ of the first PC (*p*_*1*_) of C defining the ES was calculated as defined in Equation 2:


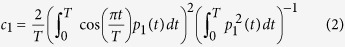


where *T* is the total simulation time.

The RMSIP index, a measure of similarity between the ES describing each replica and concatenated trajectory^49^, was computed as defined in Equation 3:


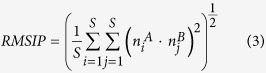


where *n*^*A*^_*i*_ and *n*^*B*^_*j*_ are the eigenvectors of the ES of the replicas *A* and *B* to be compared and *S* is the dimensionality of the ES (S=20 in this case).

### RMSD matrices and cluster analysis

The average structure for each concatenated trajectory was computed. For each frame of the concatenated trajectory the Cα -RMSD with respect to the average structure was calculated and the average RMSD value (ranging between 1.72 and 2.32 Å in all the tested GCAP1 states) was adopted as the cutoff for the cluster analysis.

Cα-RMSD matrices were computed with respect to the reference average structure defined above to perform cluster analysis of the concatenated trajectories. In order to reduce matrix dimensionality (originally 100,000 × 100,000 frames), 1 out of 4 frames of the concatenated trajectories were considered for the computation of the matrix. RMSD matrices were then processed using the Linkage^50^ and Gromos^51^ clustering algorithms, using the aforementioned average RMSD value as the cutoff. The Linkage and Gromos algorithms gave different results in terms of number of identified clusters. The Linkage algorithm identified only one cluster, at odds with the Gromos algorithm, which identified 20 to 38 clusters, depending on the state. Since the most populated cluster identified by Gromos algorithm always contained the cluster centers identified by Linkage algorithm, these latter only were used for the analyses done by WebPSN and for structural representations in Figs 3, 4, 5 and 7.

### PyInteraph

Concatenated trajectories were subjected to a dynamic network analysis provided by PyInteraph^52^, a software that generates a graph of intramolecular connections based on different types of interactions, namely salt bridges, H-bonds and hydrophobic interactions. The software computes an index of persistence of interaction based on the percentage of frames, for which distance cutoff constraints for the specific interaction are satisfied. Parameters for hydrophobic and electrostatic interactions for ions and the myristoyl group were generated manually and are available upon request. The cutoffs for distance and angle of H-bonds were set to default values (3.5 Å and 120° respectively), as well as that for electrostatic interactions (4.5 Å). The cutoff for the distance between the center of mass of the hydrophobic residue-side chains (including the myristoyl group) was set to 5.5 Å; the mass for each single atom was defined according to the CHARMM27 force field. The size of largest hydrophobic connected component (cluster) is the most restrictive constraint for the creation of the PSN^53^, therefore its value was calculated at 5% persistence intervals to determine the significance threshold for interaction persistence *p*_*T*_, which was computed as shown in Supplementary Figure S3. The *p*_*T*_ parameter was calculated for each GCAP1 simulated state (Supplementary Figure S3) and used to filter all three respective interaction graphs (e.g., electrostatic, H-bonds and hydrophobic), which were then joined in a single PSN representing the specific GCAP1 state.

### Network analysis representation

Selective betweenness (SB) was computed based on the definition of betweenness, taking into account only the shortest paths between residues *A* and *B* (*σ*_*AB*_) and not all possible shortest paths between all nodes of the PSN. In detail, SB was defined as in Equation 4:


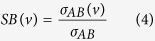


where *σ*_*AB*_(*v*) is the number of shortest paths between residues *A* and *B* that pass through residue *v*.

### WebPSN

The cluster centers identified for each GCAP1 simulated state were used as reference structures for the analysis performed by the WebPSN server^54^. The software employs an Elastic Network Model – Normal Mode Analysis (ENM-NMA) strategy to generate a PSN to investigate network properties of macromolecules. ENM-NMA was computed based on Cα atoms of the residues, Ca^2+^ and Mg^2+^ ions and the carbonyl C of the myristic group using 50 modes and Kovacs potential. The normalization factors for ions and the myristoyl group were computed based on the pdb structure, the distance cutoff for PSN calculation was set to 4.5 Å. Relationship between adjacent residues and N- and C-terminal residues were excluded and the minimum degree for the definition of a hub residue was set to 3.

## Additional Information

**How to cite this article**: Marino, V. and Dell’Orco, D. Allosteric communication pathways routed by Ca^2+^/Mg^2+^ exchange in GCAP1 selectively switch target regulation modes. *Sci. Rep.* **6**, 34277; doi: 10.1038/srep34277 (2016).

## Supplementary Material

Supplementary Information

Supplementary Video V1

Supplementary Video V2

Supplementary Video V3
