# Supplementary material for: Allosteric communication pathways routed by Ca2+/Mg2+ exchange in GCAP1 selectively switch target regulation modes
Source: Sci Rep. 2016 Oct 14;6:34277. doi: 10.1038/srep34277 (PMC5064319; doi:10.1038/srep34277)
Supplement: Supplementary Information [file srep34277-s1.pdf]

Supplementary Information

Allosteric communication pathways routed by Ca^{2+}/Mg^{2+} exchange in GCAP1 selectively switch target regulation modes

by Valerio Marino and Daniele Dell'Orco

Supplementary Tables

Supplementary Table T1. Hubs for the PSN of each mGCAP1 state generated by PyInteraph. Residues associated with cone, cone-rod and macular dystrophies are underlined and in bold

Deg	EF2^{Ca}EF3^{Ca}EF4^{Ca}	EF2^{Mg}	EF2^{Mg}EF3^{Mg}
**8**	D64 **D100**	W21 D64	D64 A78 **D100** D175
**7**	W21 R40 F96 D144 V160	R40 A78 F96 **D100** D175	W21 R40 D72 F96 I116 I122 V139 L166 L176
**6**	A52 F60 D72 E111 I119 **I143** D163 L166 D175	A52 F60 I116 I119 F135 V139 D144 V160 D163 L176	V10 A52 F60 E75 A79 I119 F135 D144 V160 D163 L183
**5**	V10 F25 L45 V56 M59 F73 E75 A78 A79 K91 L92 Y95 I122 V139 L170 L176 V180	V10 F25 V56 M59 E61 D72 F73 E75 A79 **L84** K91 L92 R93 Y95 R109 I122 S126 F156 L166 T171	F25 V56 M59 E61 F73 L92 W94 Y95 **Y99** D108 R109 E111 112L I115 R120 A121 S126 F156 L170 R178 I179 V180 R181
**4**	L13 C29 F43 S53 Y55 E57 D68 L82 **L84** R93 W94 **Y99** D108 R109 T114 I115 I116 R120 A121 S126 F135 F140 D148 **E155** F156 I157 D168 T171 I179 R182 L183 Ca1 Ca2	E11 L13 E17 Y22 C29 L36 F39 F43 L45 S53 Y55 E57 F63 F65 D68 L80 L82 V83 W94 T114 A121 A132 F140 L153 I157 D168 L170 R172 R178 R181 L183	M5 E6 L13 Y22 C29 E38 F43 L45 S51 S53 Y55 E57 F63 F65 N66 D68 V77 L80 L82 V83 **L84** K91 R93 **N104** **I107** T114 M130 A132 F140 **I143** I157 D168 T171 R172 R182 Mg1 Mg2

Supplementary Table T2. Hubs for the PSN of each nmGCAP1 state generated by PyInteraph. Residues associated with cone, cone-rod and macular dystrophies are underlined and in bold

Deg	$EF2^{Ca}EF3^{Ca}EF4^{Ca}$					$EF2^{Mg}$					$EF2^{Mg}EF3^{Mg}$				
8	W21	D64	A78	**D100**	L183	W21	D64				W21	D64			
7	F96	I122	**I143**	D144	V160	A78	A79	**D100**			A78	**D100**	V139		
	L166														
6	V10	F39	A52	F60	D72	A52	F60	E75	F96	I116	V10	R40	A52	M59	F60
	E75	K91	L92	Y95	E111	I119	I122	V160	D163		E75	A79	I119	I122	V160
	I119	F156	D163	D175											
5	M5	F25	R40	Y55	V56	V10	E11	F25	M59	D72	V56	D72	F96	R109	E111
	M59	E61	A79	L82	S126	L92	R109	S126	F135	V139	S126	F140	D144	D163	L170
	V139	**E155**	L170	L176	I179	D144	L166	D175	R178	I179	D175	R178	V180	L183	
	V180	R182				R181									
4	E6	E11	L13	C29	L36	E17	L36	F39	R40	V56	T16	E17	F25	S53	Y55
	F42	F43	S49	D68	F73	E61	F63	F65	D68	F73	N66	D68	F73	Y76	**L84**
	V77	L80	V83	K85	R93	L80	V83	**L84**	K91	W94	K85	K91	L92	**I107**	D108
	W94	D108	R109	T114	I115	Y95	T114	R120	A132	F156	T114	I115	I116	A121	F135
	I116	A121	R120	F135	F140	I157	L170	R172	L174	V180	F156	I157	L166	D168	R172
	D148	**L151**	I157	D168	T171	L183	Mg1				I179	R181	Mg1		
	R172	S173	R178	R181	Q184										
	Ca1	Ca2													

Supplementary Table T3. Hubs for the PSN of each nmGCAP1 state generated by WEBPSN. Residues associated with cone, cone-rod and macular dystrophies are underlined and in bold

mGCAP1

Deg	EF2^{Ca} EF3^{Ca} EF4^{Ca}	EF2^{Mg}	EF2^{Mg} EF3^{Mg}
6	E111	E75	E75
5	F73 E75 **E155**	F25 D68 F73 L80 Y95	W21 N66 Y76 **I107** E111
4	W21 Y22 F43 D64 N66 D68 W94 I122 **N104** **I107** D148 **L151** Ca1 Ca2	**Y99** Y22 F39 F63 D64 N66 Y76 R109 F135 Mg1	F42 F43 T62 D64 **N104** V139

nmGCAP1

Deg	EF2^{Ca} EF3^{Ca} EF4^{Ca}	EF2^{Mg}	EF2^{Mg} EF3^{Mg}
8		F42	
7		Y76	
6		W21 F39 F63 L92 L166	E111
5	W21 E111 **E155**	F25 **L84** F135 I179	N66 E75 F135
4	F25 F39 F42 D68 E75 Y76 Y95 **I143** D148 V160 Ca1	L34 F43 Y55 T62 D64 D68 E75 L80 L82 V83 R109 L153 I157 V160 L170	L34 D64 D68 I71 V83 Y95 **Y99** **N104** L112 F140 L174 Mg1

Supplementary Figures

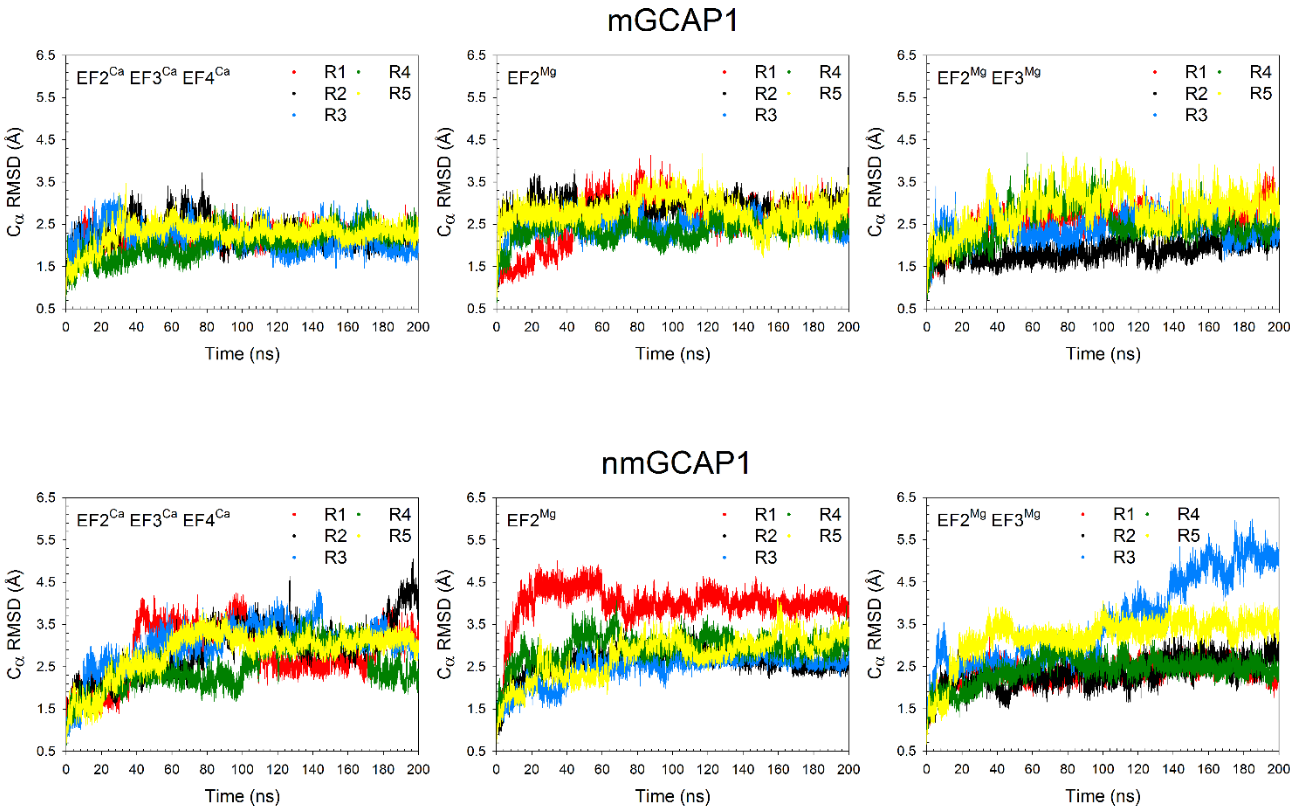

Supplementary Figure S1. Time evolution of the C_{α} RMSD of the five 200 ns MD simulation replicas (R1 to R5) calculated with respect to the relative equilibrated structure of mGCAP1 (top panels) and nmGCAP1 (bottom panels) in their $EF2^{Ca}EF3^{Ca}EF4^{Ca}$ (left), $EF2^{Mg}$ (center) and $EF2^{Mg}EF3^{Mg}$ (right) forms. R1 RMSD is represented in red, R2 RMSD is black, R3 RMSD is blue, R4 RMSD is green, R5 RMSD is yellow.

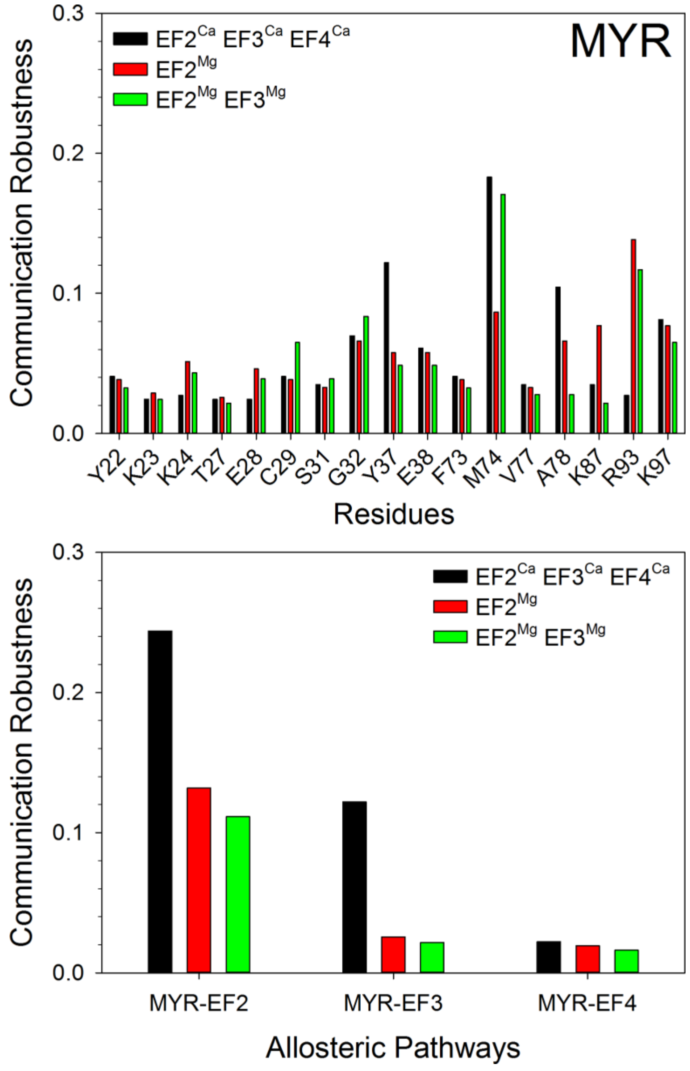

Supplementary Figure S2. Communication Robustness between the myristoyl group and residues belonging to the GC binding interface (top) and bidentate Glu residues (bottom) of EF2, EF3 and EF4 of mGCAP1 in its $EF2^{Ca}EF3^{Ca}EF4^{Ca}$ (black), $EF2^{Mg}$ (red) and $EF2^{Mg}EF3^{Mg}$ (green) forms.

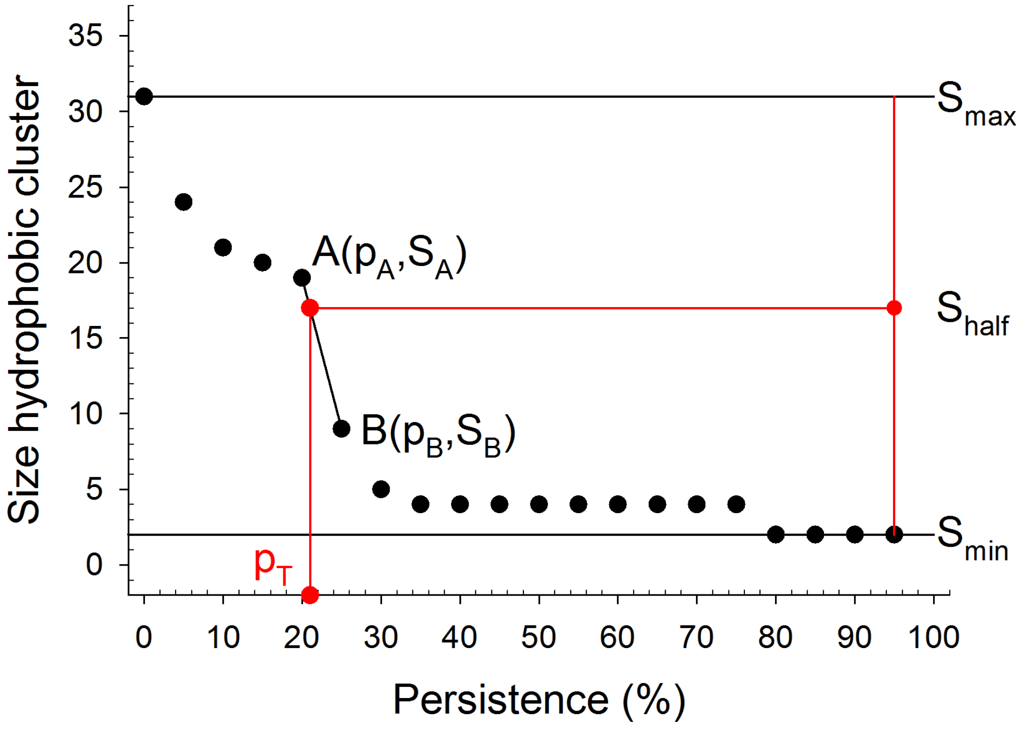

Supplementary Figure S3. Quantitative assessment of the persistence threshold p_T for PSN analyses. S_{max} and S_{min} are, respectively, the maximum and the minimum size of the largest hydrophobic cluster detected over 5% persistence intervals. S_{half} is half the difference between S_{max} and S_{min} and p_T is the calculated x value corresponding to S_{half}, derived from the linear interpolation between $A(p_A, S_A)$ and $B(p_B, S_B)$, where $[S_A, S_B]$ is the smallest 5% persistence interval containing S_{half}.

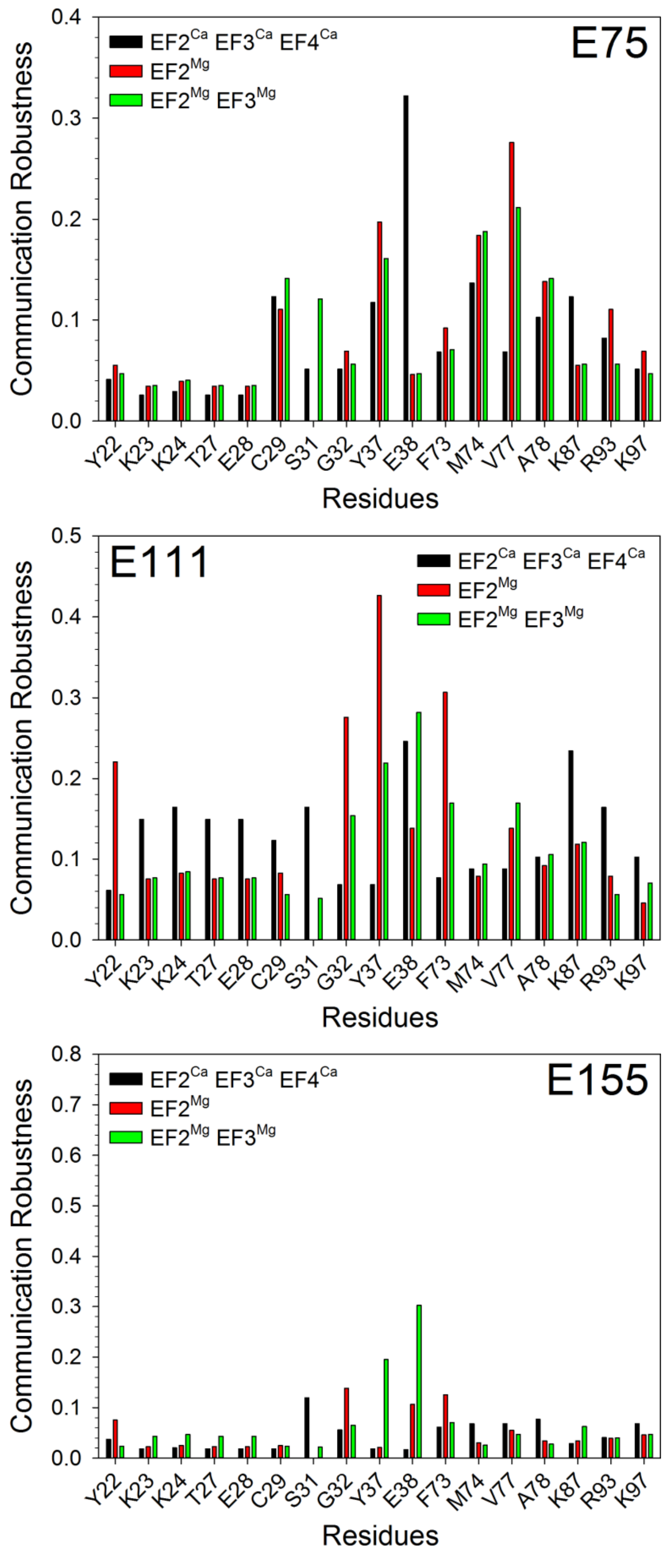

Supplementary Figure S4. Communication Robustness between bidentate Glu residues of EF2 (top), EF3 (middle), EF4 (bottom) and residues belonging to the GC binding interface of nmGCAP1 in its $EF2^{Ca}EF3^{Ca}EF4^{Ca}$ (black), $EF2^{Mg}$ (red) and $EF2^{Mg}EF3^{Mg}$ (green) forms.

Supplementary Videos

Supplementary Video V1: Paths constituted by nodes with the highest selective betweenness connecting bidentate Glu residues of EF2 and the residues belonging to the GC binding interface with the highest CR. Secondary structure is represented in grey cartoons, Ca^{2+} ions are shown as red spheres, the myristoyl group is represented as teal sticks, Cα of the residues belonging to the paths are represented as spheres, edges are represented by sticks, the side chains of the extremes of the paths are represented as sticks. Nodes and edges specific of the $EF2^{Ca}EF3^{Ca}EF4^{Ca}$ form are represented in yellow, those specific of the $EF2^{Mg}$ form are represented in blue, those in common are represented in green.

Supplementary Video V2: Paths constituted by nodes with the highest selective betweenness connecting bidentate Glu residues of EF3 and the residues belonging to the GC binding interface with the highest CR. Secondary structure is represented in grey cartoons, Ca^{2+} ions are shown as red spheres, the myristoyl group is represented as teal sticks, Cα of the residues belonging to the paths are represented as spheres, edges are represented by sticks, the side chains of the extremes of the paths are represented as sticks. Nodes and edges specific of the $EF2^{Ca}EF3^{Ca}EF4^{Ca}$ form are represented in yellow, those specific of the $EF2^{Mg}$ form are represented in blue, those in common are represented in green.

Supplementary Video V3: Paths constituted by nodes with the highest selective betweenness connecting bidentate Glu residues of EF4 and the residues belonging to the GC binding interface with the highest CR. Secondary structure is represented in grey cartoons, Ca^{2+} ions are shown as red spheres, the myristoyl group is represented as teal sticks, Cα of the residues belonging to the paths are represented as spheres, edges are represented by sticks, the side chains of the extremes of the

paths are represented as sticks. Nodes and edges specific of the $EF2^{Ca}EF3^{Ca}EF4^{Ca}$ form are represented in yellow, those specific of the $EF2^{Mg}$ form are represented in blue, those in common are represented in green, residues whose mutations are associated with Cone, Cone-Rod or Macular Dystrophies are represented in orange.
